# Mechanism of Calcium Permeation in a Glutamate Receptor Ion Channel

**DOI:** 10.1021/acs.jcim.2c01494

**Published:** 2023-02-09

**Authors:** Florian Karl Schackert, Johann Biedermann, Saeid Abdolvand, Sonja Minniberger, Chen Song, Andrew J. R. Plested, Paolo Carloni, Han Sun

**Affiliations:** ‡ Computational Biomedicine (IAS-5/INM-9), Forschungszentrum Jülich GmbH, 52428 Jülich, Germany; † Department of Physics, RWTH Aachen University, 52062 Aachen, Germany; ¶ Institute of Biology, Cellular Biophysics, Humboldt Universität zu Berlin, 10115 Berlin, Germany; § Leibniz Forschungsinstitut für Molekulare Pharmakologie, 13125 Berlin, Germany; ∥ Center for Quantitative Biology, Academy for Advanced Interdisciplinary Studies, Peking University, Beijing, 100871, China; ⊥ Peking-Tsinghua Center for Life Sciences, Academy for Advanced Interdisciplinary Studies, Peking University, Beijing, 100871, China; # Institute of Chemistry, TU Berlin, Straße des 17 Juni 135, 10623 Berlin, Germany

## Abstract

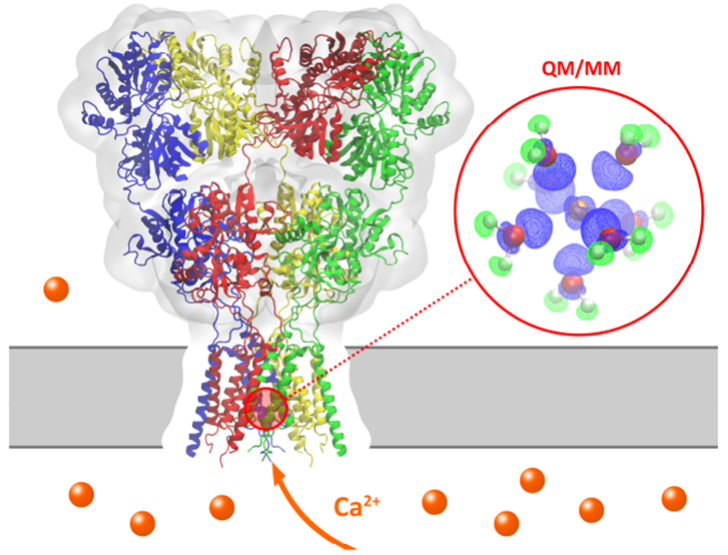

The α-amino-3-hydroxy-5-methyl-4-isoxazolepropionic acid receptors (AMPARs) are neurotransmitter-activated cation channels ubiquitously expressed in vertebrate brains. The regulation of calcium flux through the channel pore by RNA-editing is linked to synaptic plasticity while excessive calcium influx poses a risk for neurodegeneration. Unfortunately, the molecular mechanisms underlying this key process are mostly unknown. Here, we investigated calcium conduction in calcium-permeable AMPAR using Molecular Dynamics (MD) simulations with recently introduced multisite force-field parameters for Ca^2+^. Our calculations are consistent with experiment and explain the distinct calcium permeability in different RNA-edited forms of GluA2. For one of the identified metal binding sites, multiscale Quantum Mechanics/Molecular Mechanics (QM/MM) simulations further validated the results from MD and revealed small but reproducible charge transfer between the metal ion and its first solvation shell. In addition, the ion occupancy derived from MD simulations independently reproduced the Ca^2+^ binding profile in an X-ray structure of an NaK channel mimicking the AMPAR selectivity filter. This integrated study comprising X-ray crystallography, multisite MD, and multiscale QM/MM simulations provides unprecedented insights into Ca^2+^ permeation mechanisms in AMPARs, and paves the way for studying other biological processes in which Ca^2+^ plays a pivotal role.

## Introduction

Ionotropic glutamate receptors are cation-permeable ion channels responsible for fast excitatory signal transmission in vertebrate neurons.^1^ Upon glutamate binding, the transmembrane channel pore adopts an open configuration that is nonselective for monovalent cations. In the α-amino-3-hydroxy-5-methyl-4-isoxazolepropionic acid receptor (AMPAR), the permeability for calcium depends exquisitely on its composition. The GluA2 subunit gene encodes a Gln at residue 586, but post-transcriptional RNA editing yields an Arg residue (Q/R site) at about 96%^2^ of subunits in the brain. If they lack the edited GluA2(R) subunit, AMPA receptors are readily Ca^2+^ permeable.^3^ In principal neurons, most AMPARs contain GluA2 and are thus calcium impermeable, whereas in interneurons, GluA2 is broadly absent.^4^ Calcium flux through AMPARs is linked to synaptic plasticity through transient insertion of calcium-permeable GluA1 homomers,^5^ while excessive calcium influx in the absence of ADAR2 editing of the GluA2 subunit represents a risk for neurodegeneration.^6^

**Figure 1:**
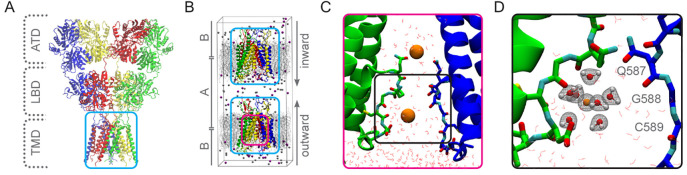
(A) AMPARs (PDB ID: 5WEO) consist of an Amino Terminal Domain (ATD), a Ligand Binding Domain (LBD), and a Transmembrane Domain (TMD). (B) The TMD and linkers to the LBD (cyan box) were included in the computational electrophysiology simulations,^7^ where the transmembrane potential was generated by introducing an ion imbalance between compartment A and B. (C) A snapshot of GluA2(Q) selected from the MD simulations showing the selectivity filter region and coordinating Ca^2+^ ions (orange spheres). Water molecules are represented by lines, protein in cartoon with key residues in sticks. For better visibility, only two opposing subunits are shown. (D) A selected QM/MM snapshot. Atoms that are included in the QM partition (Ca_aq_^2+^) are shown in ball and stick with their electron density (isovalue 0.1 *e*/*a*_0_^3^) in gray wireframe.

Recent cryo-EM and X-ray studies of several AMPAR isoforms in closed, partially open, and fully open states^8−11^ have revealed structural determinants of gating (Figure 1A). Yet, the details of calcium permeation mechanisms remain unknown. Atomistic Molecular Dynamics (MD) based on biomolecular force fields such as AMBER^12^ or CHARMM^13^ are powerful approaches to investigate the permeation mechanisms of monovalent cations in ion channels^14−22^ along with AMPAR.^23^ The latter simulations used the computational electrophysiology setup (Figure 1B), including a transmembrane potential induced by the ion concentration difference across the membrane,^7^ and revealed that monovalent ions, such as Na^+^, K^+^, and Cs^+^, permeate at similar rates through the GluA2 open pore by exploiting different binding sites and hydration states, and not by ion-dependent structural accommodations. Unfortunately, the lack of accurate force field parameters for Ca^2+^ due to errors introduced by representing its divalent charge at a single point, has made simulating calcium permeation in ion channels, including AMPARs, almost impossible.

One approach to remediate the unrealistic behavior of divalent cations in MD simulations are multisite models, pioneered by Warshel and co-workers for Mg^2+^.^24^ One such model for Ca^2+^,^25^ compatible with CHARMM,^13^ very recently allowed simulations of calcium conduction in the ryanodine receptor (RyR)^26,27^ and in transient receptor potential ion channels (TRPV).^28^ Although the calcium conductances derived from these simulations were similar to experiments, further validation of this new Ca^2+^ parametrization is essential in order to extend this approach for general use.

The goal of the paper is 2-fold: first, we provide insight on mechanistic aspects of calcium(II) ion permeation; second, we validate the new force field developed specific for calcium ions.^25^ To this aim, we investigate calcium permeation in two cation channels: The AMPA receptor GluA2 and an AMPAR channel pore mimic that incorporates the partial selectivity filter (SF) sequence of AMPAR into a bacterial NaK channel. The latter approach was successfully employed in the past to study ion binding and Ca^2+^ block in the cyclic nucleotide-gated channel pore.^29−31^ We simulated Ca^2+^ permeation (using computational electrophysiology) in the GluA2 channel, showing Ca^2+^ binding sites in the SF region that are distinct from those of monovalent cations. A stable Ca^2+^ binding site identified from MD could be verified by Quantum Mechanics/Molecular Mechanics (QM/MM) simulations (Figure 1C/D), with matching Ca^2+^ hydration statistics. Critically, QM/MM simulations also revealed small but reproducible charge transfer between the metal ion and its first solvation shell. The validity of the multisite model was further confirmed with experimental data from a high-resolution X-ray structure of the AMPAR pore mimic with Ca^2+^ because the observed Ca^2+^ binding profile was fully reproduced by the multisite Ca^2+^ model in MD simulations.

## Results

### Calcium Permeation Mechanism in AMPA Receptors Revealed by MD Simulations

We employed the computational electrophysiology setup to simulate Ca^2+^ permeation across the unedited form of GluA2, a representative model of a calcium-permeable AMPAR. A cryo-EM structure of an open conformation of GluA2 (PDB ID: 5WEO(9)) was used as a starting point for the MD simulations. All auxiliary proteins as well as the amino-terminal domain (ATD) and the ligand binding domain (LBD) were removed, while we held the transmembrane domain (TMD) open by physically restraining the end of the truncated linkers. Within six runs of 250 ns simulation time, we observed persistent outward Ca^2+^ permeation in each simulation run, leading to a conductance of (35 ± 18) pS, that is comparable with previously simulated K^+^ conductance using a similar setup.^23^ Like monovalent cations, Ca^2+^ follows a loosely coupled knock-on mechanism,^23,28^ where in most cases the exit of an ion to the upper cavity is closely followed by the entry of an ion in the SF (Movie S1).

**Figure 2:**
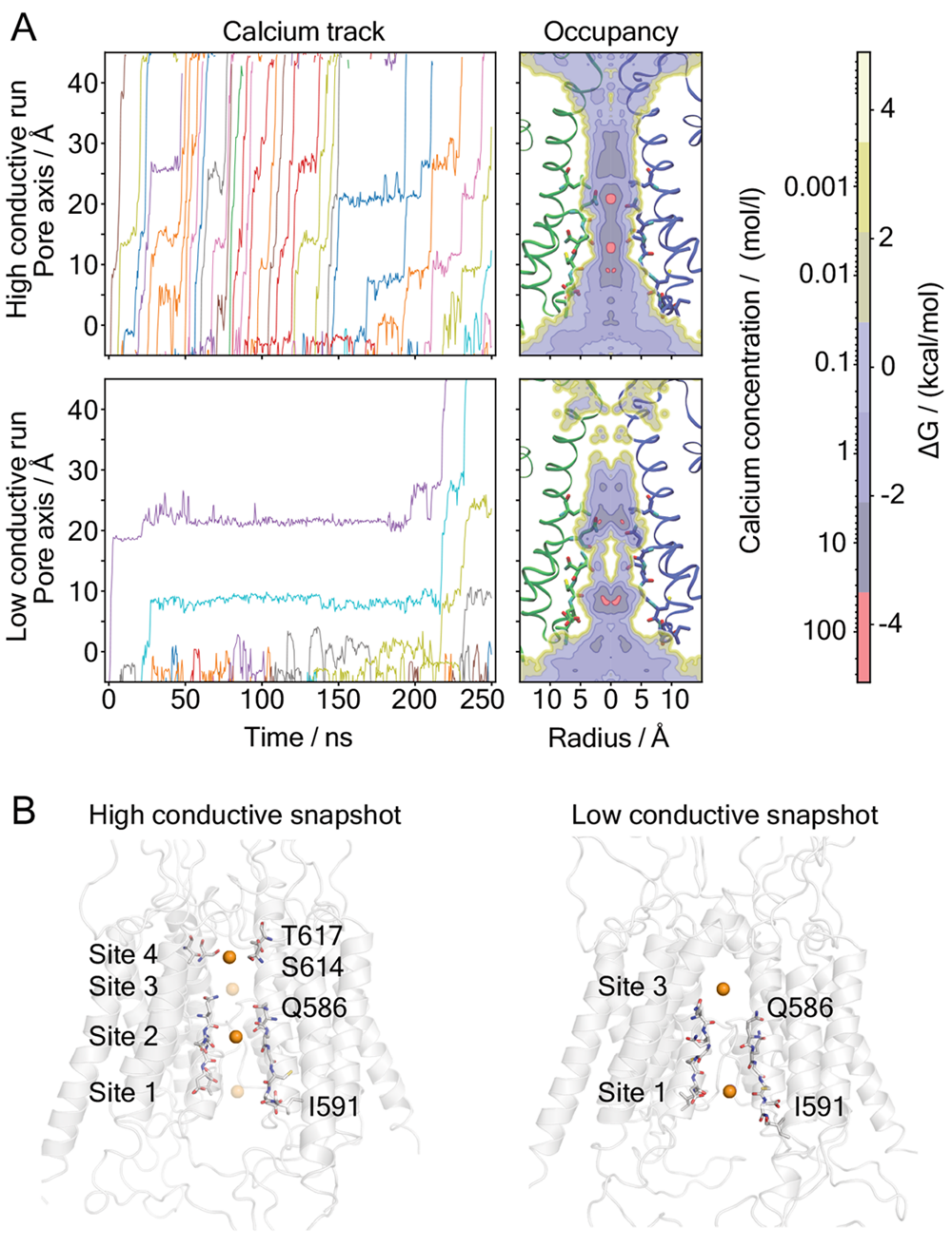
(A) Representative traces of Ca^2+^ passing through the SF of the GluA2(Q) channel pore during a “high conductive” (top) and a “low conductive” MD simulation run (bottom). The cross-section of the GluA2 transmembrane domain is shown in the second column together with the two-dimensional Ca^2+^ occupancy derived from MD. The latter is plotted on a logarithmic scale as concentration, and linearly as free energy. (B) Selected snapshots of “low” and “high” conductive MD simulations revealing the major Ca^2+^ binding sites within and above the SF. In the selected “high conductive” snapshot, only Ca^2+^ at sites 2 and 4 are present simultaneously, while two modeled ions at sites 1 and 3 are displayed in transparent spheres.

Intriguingly, we found large variations in the conductance across different simulation replicas (Figure 2A). These “high conductive” and “low conductive” runs differ in their preferable Ca^2+^ binding sites in the channel. In high conductive runs, Ca^2+^ occupies four sites (Figure 2B): **Site 1**, located in the lower SF region. Here, Ca^2+^ may be coordinated by G588, C589, and D590 backbone atoms. **Site 2** is close to the Q/R site, corresponding to the main monovalent cation binding site determined from MD^23^ and cryo-EM^9^ (Figure S1). The metal ligands include the Q586 and Q587 backbone atoms as well as the Q586 side chain; **Site 3**, located slightly above the SF. Here, the only protein residue interacting with Ca^2+^ is the Q586 side-chain. **Site 4**, located in the cavity between the SF and the upper helix bundle gate. S614 and T617 backbones and side chains may coordinate the metal ion. In contrast to high conductive runs, only **Site 1** and **Site 3** remain in low conductive runs. Thus, in high conductive runs, Ca^2+^ ions pause frequently but briefly at more sites along the ion conduction pathway, which substantially reduces the energy barriers between the two principal binding sites observed in the low conductive simulations.

Higher voltages (around 600 mV) compared to physiology-relevant conditions were applied to accelerate the sampling of ion permeation events in the nanosecond to microsecond time scale. This setup further allowed us to compare the results of Ca^2+^ conduction with our previous monovalent cation permeation simulations in AMPAR under similar conditions.^23^

### Hydration Statistics of Calcium(II) in AMPAR Are Congruent between MD and QM/MM Simulations

**Figure 3:**
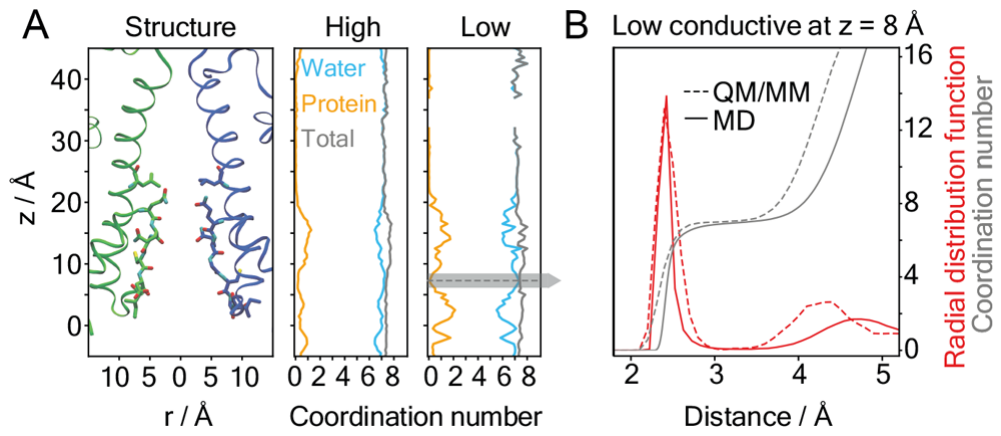
(A) Number of coordinated water and protein residues during calcium permeation through the channel pore for high and low conductive runs. (B) Calcium–oxygen radial distribution function (red) and running coordination number (gray) at *z* = 8 Å. Solid/dashed lines indicate results from QM/MM/MD, respectively.

During the MD simulations, Ca^2+^ remains almost entirely hydrated in the SF with six to seven water molecules in coordination (Figure 3A). To further investigate the robustness of the multisite parameters for simulating calcium permeation in the AMPAR, we employed the recently developed, massively parallel Quantum Mechanics/Molecular Mechanics MD code MiMiC,^32^ which scales up to several thousands of cores, allowing excellent utilization of modern supercomputer architectures such as JURECA-DC.^33^ A representative snapshot of the low conductive MD simulations with a Ca^2+^ at **Site 1** served as starting structure for the QM/MM simulations. Here, Ca^2+^ and its first hydration shell were treated at the Density Functional Theory (DFT) level, while all other water molecules as well as protein and lipids were described classically (Figure 1C/D). By including the entire system simulated by classical MD (as opposed to simulating calcium in bulk water), we take into account the large electric field of the protein. Also, we describe properly the structure and dynamics of calcium-bound water molecules, which may form H-bonds to protein residues and are confined in the channel. All following properties were calculated from eight QM/MM trajectories, which correspond to a total production phase of 42 ps.

The calcium ion coordinates seven water molecules in the MD simulations, consistent with previous computational studies, which suggest that the number of water molecules binding to the metal ion ranges from six to eight.^34−41^ With our QM/MM simulations, we sample configurations with this 7-fold coordination. Ca_aq_^2+^ consists solely of QM atoms, i.e., before water exchange takes place. Furthermore, at **Site 1**, protein residues do not bind to the metal ion, neither in the MD nor in the QM/MM simulations. The calcium–oxygen distances as emerging from the Radial Distribution Functions (RDFs) are 2.4 Å in both MD and QM/MM (Figure 3B). These values are close to full *ab initio* MD studies of the ion in bulk water.^38−40^ The distributions of the second shell are rather broad with maxima at 4.4 Å for the QM/MM and at 4.7 Å for the multisite model MD (Figure 3B), as compared to an experimental value of 4.6 Å.^42^ The difference between QM/MM- and force field-based MD might be caused, at least in part, by the lack of polarization and charge transfer effects in the latter, as seen in other metal ions.^43−45^

### Sizable Polarization Effects during Permeation

**Figure 4:**
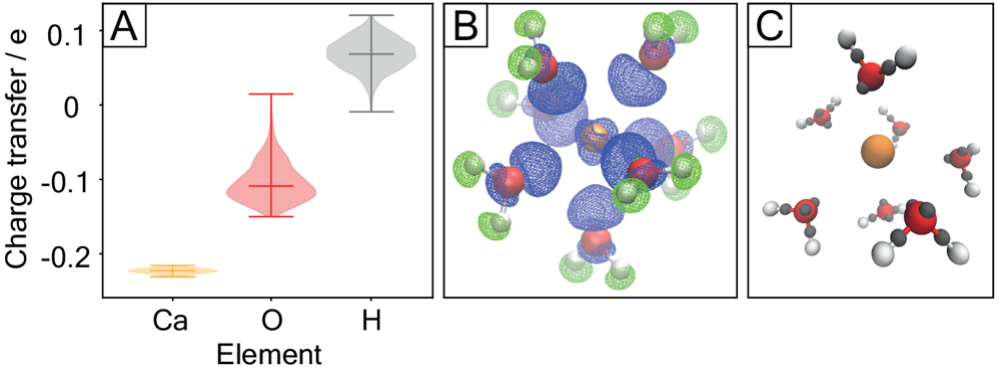
(A) Integrated charge transfer between the calcium ion and its first hydration sphere evaluated for 82 snapshots on the B3LYP level and grouped by element. (B) Electron density difference Δϱ between the presence and absence of Ca^2+^ for one QM/MM snapshot. Blue/green surfaces represent an isovalue of +0.005/ – 0.005 *e*/*a*_0_^3^, respectively. (C) Maximally localized Wannier functions of coordinated water molecules (gray dots) represent oxygen lone pairs and O–H bonds.

From the QM/MM simulations, we further investigated the polarization effects of Ca^2+^ during permeation. We found that each water molecule binding to the metal ion donates on average 0.03 electrons. This leads to an effective charge of the calcium ion of 1.8 *e*. The water molecules themselves are more polarized when they are coordinated by the calcium ion. Oxygen atoms attract additional (0.10 ± 0.03) electrons from the hydrogen atoms, while the hydrogen atoms lose (0.07 ± 0.02) *e* (Figure 4A).

Upon calcium binding, the maxima of the localized Wannier functions (Figure 4C) representing the oxygen electron lone pairs shift toward the metal ion by (0.03 ± 0.01) Å, while the ones representing the O–H bonds shift by (0.06 ± 0.01) Å toward the oxygen, making the O–H bonds more polar. This is expected to strengthen the hydrogen bonds with the surrounding water molecules, consistently with the experimental evidence that water molecules are more strongly hydrogen bonded in the presence of Ca^2+^ than without.^46^ The resulting dipole moment of the water molecules is (2.9 ± 0.2) D in the presence of Ca^2+^ and (1.9 ± 0.3) D without the metal ion, to be compared to that in bulk water ranging from (2.9 ± 0.3) D to (3.2 ± 0.3) D, depending on the exchange-correlation functional.^47^

### Calcium Binding Profile in an AMPAR Pore Mimic

**Figure 5:**
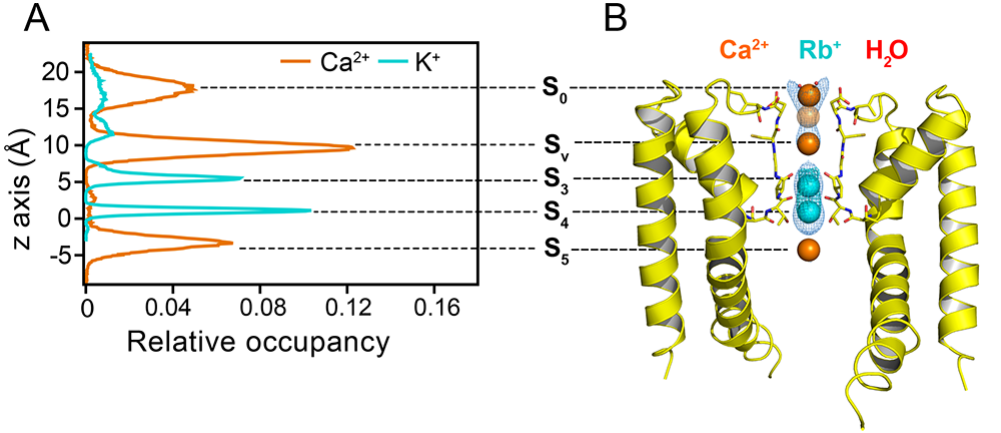
(A) Relative one-dimensional ion occupancy in the SF of NaK C-DI derived from Ca^2+^ MD simulations (orange), performed with the multisite Ca^2+^ model compatible with CHARMM (this work) and K^+^ MD simulations (cyan), with CHARMM.^48^ The origin is set to the hydroxy group of T63. (B) The 2F_o_–F_c_ electron density maps (blue mesh, contoured at 1σ) around Ca^2+^ (orange), Rb^+^ (cyan) and water molecules (red) along the ion channel path of NaK C-DI together with the anomalous difference density of Rb^+^ (cyan mesh), contoured at 3σ.

To independently assess the accuracy of the predictions obtained using the recently developed force field for the calcium ions, we obtained experimental structural information on the AMPAR selectivity filter in the presence of Ca^2+^. We used the prokaryotic NaK channel as scaffold for crystallization, as in our previous study of monovalent cations.^48^ Specifically, we determined the crystal structures of a construct that replaces DGNF in the SF of NaK with C-DI from AMPAR, in the presence of either Rb^+^ and Ca^2+^ (resolution: 2.75 Å, PDB ID: 8AYQ, Figure 5B) or Rb^+^ and Ba^2+^ (resolution: 2.10 Å, PDB ID: 8AYP, Figure S4).

The X-ray structures revealed distinct ion binding profiles for monovalent and divalent cations within and around the SF (Figure 5B). The monovalent cations bind at sites S_3_ and S_4_ in the lower part of the SF formed by TTV. This is consistent with previous X-ray analysis of a NaK C-DI mutant with monovalent cations only.^48^ In contrast, the Ca^2+^ density is observed at three distinct sites (Figure 5B): (i) S_5_: directly below the SF formed by the T63 side chain; (ii) S_v_: in the vestibule, stabilized by the C66 backbone; (iii) S_0_: at the upper mouth of the SF bonded to the D67 side chain.

Starting from the currently determined X-ray structure of NaK C-DI, we performed MD simulations to simulate Ca^2+^ permeation following the same approach as for native AMPAR. We observed over 250 outward Ca^2+^ permeation events over 11 μs simulations (Figure S5). Most importantly, the Ca^2+^ occupancy derived from these simulations is in excellent agreement with the X-ray data, showing distinct ion occupation at three sites: S_0_, S_v_, and S_5_ (Figure 5A). Similar to the simulations of AMPAR, Ca^2+^ ions remain hydrated in the SF during permeation in NaK C-DI (Figure S6).

## Discussion

We have presented an integrated study using X-ray crystallography, MD and QM/MM simulations revealing a detailed calcium conduction mechanism in the vertebrate AMPAR channel pore and demonstrating polarization effects of calcium during conduction. Previous work suggests that Ca^2+^ permeates through calcium-permeable AMPARs at similar rates as monovalent cations.^3,49^ The employed multisite model force field parameters for Ca^2+^ resulted in a conductivity that is similar to our monovalent cation simulations,^23^ therefore approximately reproducing these wet experimental results with unbiased MD simulations. The calcium conduction mechanism derived from MD simulations using the recently introduced Ca^2+^ parametrization was further verified by two orthogonal approaches: (i) comparison between the experimentally determined and simulated Ca^2+^ binding profiles in an AMPAR pore mimic; (ii) high-level QM/MM simulations of Ca^2+^ binding in native AMPAR.

Regarding (i), we identified multiple Ca^2+^ binding sites along the ion conduction pathway. Especially one major Ca^2+^ binding site that is only attributed to the side-chain of Q586 at the Q/R editing site is rather weak in the monovalent cation simulations (Figure S1). Editing of the Gln to a positively charged Arg is therefore expected to drastically decrease the affinity of Ca^2+^ for this site, consistent with the experimentally observed calcium nonpermeability of the GluA2(R) edited form.^3,50,51^ Furthermore, our MD simulations revealed a low and a high conducting mode that exhibit distinct preferential Ca^2+^ binding sites in the channel. It is possible that the low-conductive mode relates to a channel block by permeant Ca^2+^, because experimentally it was shown that Ca^2+^ ions tend to slow the passage of copermeant monovalent cations.^3^ This hypothesis is supported by the notable differences in binding sites between monovalent and divalent cations. While monovalent cations prefer binding sites at a narrow pore, i.e., the TTV filter in NaK C-DI^48^ and the QQ filter in AMPAR,^23^ Ca^2+^ ions occupy more sites along the SF with substantial presence also at the wide CDI part in both NaK C-DI and AMPAR. By occupying more sites along the ion conduction pathway, calcium could easily block copermeant monovalent cations that exhibit half of its valence. Indeed, in NaK C-DI calcium and barium reside stably at a single site within the wide section of the selectivity filter, which shows in contrast no occupancy by monovalent ions in the presence of divalent ions. Nonetheless, we can not establish the molecular determinants of these two different conducting states so far and hope to provide more mechanistic insights in a future study by a combination of Markov state modeling^52^ and functional validation. Furthermore, Ca^2+^ ions are persistently hydrated during permeation. In contrast, monovalent cations lose and regain waters from their first shell in a dynamic fashion. This observation is similar to calcium permeation simulations in RyR receptors, where Ca^2+^ is fully hydrated during the entire conduction. The pore of RyR is however substantially wider than that of AMPAR (narrowest part of the SF about 7 Å in AMPAR^9^ and 10 Å in RyR^53^).

As for (ii), our QM/MM simulations revealed sizable polarization of the hydration shell along with small charge transfer to Ca^2+^, within the well-known limitation of standard DFT functionals to overestimate charge transfer^54^ and the relatively short time-scale of the simulations. The resulting polarization of the bound water likely impacts the hydrogen bond network of hydrated Ca^2+^ in the channel, further suggesting that conductance derived from simulations with nonpolarizable force fields may be less accurate than the conductance derived from simulations that account for polarization effects.

## Conclusions

In conclusion, we show a remarkable congruence between experimental data, classical MD and *ab initio* QM/MM MD simulations in studying calcium permeation mechanism of AMPAR. Ca^2+^ ions occupy more sites along the ion conduction pathway compared to monovalent cations and its permeability can be thus controlled by single-residue editing in the selectivity filter. The hydration statistics emerging from MD and QM/MM are rather similar, especially regarding the first hydration shell. Still, QM/MM simulations revealed small but sizable polarization effects during Ca^2+^ permeation. We thus propose that the Ca^2+^ ion multisite model captures electronic structure effects fairly well and that it is suitable for studying other important biological systems where calcium plays a pivotal role. We envision that polarizable force fields^55−58^ or potentials derived from machine learning^59^ could enable even more accurate simulations of calcium permeation in AMPARs and other ion channels.

## Data and Software Availability

The structure of the AMPAR receptor channel was obtained from the PDB (www.rcsb.org). The membrane model was built with the CHARMM-GUI web server (charmm-gui.org). Force field-based molecular dynamics were carried out with the GROMACS 2019 software suite (www.gromacs.org). For the QM/MM simulations, we used the MiMiC framework (www.mimic-project.org). This employs GROMACS 2020 and CPMD v4.3 (www.cpmd.org). Pseudopotentials are available for download from the CPMD Web site after registration.

X-ray data processing and scaling was performed with XDSAPP. Refinement and manual model building were performed in Phenix (www.phenix-online.org) and Coot (www2.mrc-lmb.cam.ac.uk/personal/pemsley/coot/), respectively. Structures have been deposited in the PDB with accession numbers 8AYQ (Rb^+^ with Ca^2+^) and 8AYP (Rb^+^ with Ba^2+^).

Representations were created using VMD 1.9.3 (www.ks.uiuc.edu/Research/vmd/) and PyMol (https://pymol.org/2/). The parameter files used here, the input files, and initial atomic coordinates are deposited in the Open Science Framework (https://osf.io) and are assigned the DOI 10.17605/OSF.IO/3SRV6.

## Methods

### Protein Crystallization and Structure Determination

Protein expression and purification were performed as previously described.^48^ The hexahistidine tag was removed by incubation with trypsin (1:250 molar ratio) for 1 h at room temperature. Afterward, the protein was run on a Superdex 200 increase (10/300) column in 20 mM HEPES (NaOH) pH 7.5, 5 mM DM, 100 mM RbCl, and 10 mM CaCl_2_ or BaCl_2_. Purified protein was concentrated to 12 mg/mL and crystallized with the sitting drop vapor diffusion method at 20 °C by mixing equal volumes of protein and reservoir solution (40% (v/v) (±)-2-methyl-2,4-pentanediol (MPD) and 100 mM 2-morpholin-4-ylethanesulfonic acid (MES) - NaOH pH 6). Crystals were directly flash-frozen in liquid nitrogen without additional cryoprotection. X-ray diffraction data were collected at 100 K at beamline BL14.1 at BESSY II (Berlin, Germany)^60^ at 15.205 keV to record the anomalous signal of Rb^+^ and additionally at 13.5 keV for the Ba^2+^ complex. Both crystals belonged to spacegroup *C*222_1_ with unit cell dimensions *a* = 68 Å, *b* = 175–177 Å, *c* = 68 Å, α = β = γ = 90°. Data processing and scaling were performed with XDSAPP.^61^ The structures were solved by molecular replacement using chain A of NaKΔ19 (PDB 3E86) as a search model with the selectivity filter residues omitted. Repeated cycles of refinement and manual model building were performed in Phenix^62^ and Coot,^63^ respectively.

### Computational Electrophysiology Simulations

We used the deterministic computational electrophysiology setup, where a transmembrane potential is induced by the ion concentration difference across the membrane,^7^ to simulate Ca^2+^ permeation in GluA2 and NaK C-DI, as implemented in the simulation package GROMACS.^64^ For simulations of the open-state GluA2, we used a cryo-EM structure (PDB ID: 5WEO(9)) as the starting configuration. The currently determined X-ray structure of NaK C-DI with Ca^2+^ was employed as starting configuration for simulations of the pore mimic. Each system was embedded in a patch of a 1-palmitoyl-2-oleoyl-*sn*-glycero-3-phosphocholine (POPC) membrane and solvated with an explicit water model. For the simulations of GluA2, only the transmembrane domain and linkers to the ligand binding domain were included in the MD simulations (Figure 1, see Supporting Information for details). MD simulations with Ca^2+^ were performed with a multisite Ca^2+^ model that is compatible with the CHARMM36^13^ force field.^25^

### QM/MM Simulations

A representative snapshot of the low conductance mode observed during the force field based MD simulations served as the starting structure for our QM/MM simulations. The MiMiC interface^32,65,66^ was used to couple GROMACS^64^ and CPMD.^67^ The QM part comprises a calcium ion at **Site 1** together with its first hydration shell and was treated with plane-wave and pseudopotential based Density Functional Theory (DFT), while the MM part of the system was described by the same force field as in the classical MD simulations. The Born–Oppenheimer Molecular Dynamics (BOMD) scheme, employing the BLYP^68,69^ functional, was used with a time step of 0.5 fs to simulate the system at 303 K. A simulated annealing followed by a linear reheating was carried out before the production phase (42 ps in total, see Supporting Information for details). For the charge transfer analysis, we selected 82 snapshots from the QM/MM production run trajectories. For each snapshot, we calculated the electronic density of the complete QM region embedded in the MM surrounding (complex), the calcium ion in the gas phase (ligand), and the QM region without Ca^2+^ embedded in the MM region (rest). We used the B3LYP functional.^70^ We then calculated the difference in electron density Δϱ following
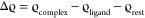
1The transferred charges were then given by integrating Δϱ over the Voronoi volume of each atom.^71^ The Maximally Localized Wannier Functions (MLWF)^47^ were calculated as well and then averaged over all snapshots. The analysis of the simulations results was performed with in-house scripts.

## Supporting Information Available

Movie depicting the exit of an ion to the upper cavity closely followed by the entry of an ion in the SF (MP4)
Computational Details for MD simulations of the AMPAR transmembrane domain. Computational Details for QM/MM simulations. Description of QM/MM solvent exchange. Electron density maps, simulated outward conductance, oxygen coordination, and data collection and refinement statistics for calcium binding in an AMPAR pore mimic (PDF)
