# Supplementary material for: Mechanism of Calcium Permeation in a Glutamate Receptor Ion Channel
Source: J Chem Inf Model. 2023 Feb 9;63(4):1293–300. doi: 10.1021/acs.jcim.2c01494 (PMC9976283; doi:10.1021/acs.jcim.2c01494)
Supplement: Supplementary file 2 — ci2c01494_si_002.pdf [file ci2c01494_si_002.pdf]

Mechanism of Calcium Permeation in a Glutamate Receptor Ion Channel Supplementary Information

Florian Karl Schackert,[†,‡,@] Johann Biedermann,[¶,§,@] Saeid Abdolvand,[¶,§] Sonja Minniberger,[¶,§] Chen Song,[‖,⊥] Andrew J. R. Plested,[¶,§] Paolo Carloni,[*,†,‡] and Han Sun[*,§,#]

† *Department of Physics, RWTH Aachen University, 52062 Aachen, Germany*

‡ *Computational Biomedicine (IAS-5/INM-9), Forschungszentrum Jülich GmbH, 52428 Jülich, Germany*

¶ *Institute of Biology, Cellular Biophysics, Humboldt Universität zu Berlin, 10115 Berlin, Germany*

§ *Leibniz Forschungsinstitut für Molekulare Pharmakologie, 13125 Berlin, Germany*

‖ *Center for Quantitative Biology, Academy for Advanced Interdisciplinary Studies, Peking University, Beijing, 100871, China*

⊥ *Peking-Tsinghua Center for Life Sciences, Academy for Advanced Interdisciplinary Studies, Peking University, Beijing, 100871, China*

Institute of Chemistry, Straße des 17 Juni 135, TU Berlin, 10623 Berlin, Germany

@*Shared First Authorship*

E-mail: p.carloni@fz-juelich.de; hsun@fmp-berlin.de

MD simulations of the AMPAR transmembrane domain

A cryo-EM structure of an open conformation of the AMPA receptor (PDB ID: 5WEO[1]) was used as a starting point for our Molecular Dynamics (MD) simulations. All auxiliary proteins as well as the amino-terminal and the ligand binding domain of the AMPA receptor were removed. The CHARMM-GUI membrane builder[2] was used to create a model system with the Trans-Membrane Domain (TMD) of the AMPA receptor embedded in a 1-Palmitoyl-2-oleoyl-sn-glycero-3-phosphocholine (POPC) bilayer. The lipid bilayer with the TMD was solvated in a 10 nm by 10 nm by 10 nm box of TIP3P water.[3] 107 mM calcium ions and 195 mM chloride ions were added, resulting in a neutral total charge of the system.

To simulate ion permeation, computational electrophysiology[4] was utilized: A copy of the system was added on top of the existing one. This double bilayer setup contains two compartments. By fixing the number of cations and anions in these compartments, a charge gradient is induced and the resulting potential drives ions through the open AMPA receptor channel. In our case, a charge imbalance of six elementary charges (three Ca^{2+}) led to a transmembrane potential of 570 mV to 750 mV during the simulations. The deterministic approach was used to exchange an ion after each ion permeation with water molecules from the opposite compartment. A split cylinder with radius 0.8 nm around the center of mass of heavy atoms from residues 586 to 590 with an extension of 0.5 nm above and 0.3 nm below describes the compartments dividing the selectivity filter. A check for ion permeation was performed every 100 integration steps.

GROMACS 2019.5[5] was used to simulate the system with the CHARMM36[6] force field. All energy minimization and equilibration steps were performed as suggested by CHARMM-GUI.[2] An energy minimization according to GROMACS steepest descent algorithm with a maximum force tolerance of 1000 kJ/mol/nm and a maximum of 5000 steps was performed. Backbone atoms are restrained with 4000 kJ/mol/nm (R_{BB}) and side-chain atoms with 2000 kJ/mol/nm (R_{SC}). Lipids were restrained in the position of phosphate (R_{LP}) and the dihedral angles of the close lipid tail carbons (R_{LD}) with 1000 kJ/mol/nm. Six equilibration steps follow, see

Table S1. With increasing integration time step and decreasing restraints the system reached an equilibrated state. All equilibration runs were carried out with a berendsen thermostat (303 K) and barostat,[7] and LINCS[8] constraints. Particle mesh Ewald[9] was used for fast calculation of long-range electrostatic interactions.

Table S1: Equilibration runs according to CHARMM-GUI. Restraints are given in kJ/mol/nm.

	δt / ps	Time / ps	Barostat	R_{BB}	R_{SC}	R_{LP}	R_{LD}
1	0.001	25	isotropic	2000	1000	1000	1000
2	0.001	25	isotropic	1000	500	1000	400
3	0.001	25	semiisotropic	500	200	400	200
4	0.002	100	semiisotropic	500	200	200	200
5	0.002	100	semiisotropic	200	50	40	100
6	0.002	100	semiisotropic	50	0	0	0

Henceforth, we used recently published multi-site model parameters for calcium.[10] A Velocity Verlet integrator with a timestep of 2 fs was used in combination with a velocity rescaling thermostat[11] with 1 ps coupling constant at 303 K and a semiisotropic Berendsen[7] barostat with 5 ps coupling constant, 1 bar reference pressure, and 4.5×10^{-5} 1/bar compressibility. Covalent bonds involving hydrogen atoms were constrained via LINCS.[8] Additional 5 ns equilibration without restraints followed by 15 ns with restraints on the protein termini were deployed before production runs. In production runs, the positions of the ends of the truncated linkers were restrained to prevent the channel from closing. Trajectories were collected for six replicas of 250 ns simulation time each. Each simulation was performed at 303 K. An imbalance of six elementary charges between the two compartments resulted in varying potential from 570 mv up to 750 mv. For the outward permeation, an average conductance of (35 ± 18) pS was obtained.

To obtain representative structures for high and low conductive states (see Figure 2B), we performed cluster analysis on the lowest (simulation 1) and highest (simulation 2) conductive classical MD simulations. The corresponding GROMACS[5] subroutine was used with the single linkage method and a 0.1 nm RMSD cutoff. Only heavy atoms of the selectivity filter residues

$_{856}$QQGDC$_{590}$ were included in the clustering. The central structure of the respective largest cluster has been chosen to represent low and high conductive states.

Table S2: Transmembrane potential (mV), number of calcium permeation events, and conductance (pS) for the six runs of 250 ns MD simulations.

Simulation	1	2	3	4	5	6
Potential	570	620	660	640	750	580
Calcium permeation events	3	28	13	16	17	26
Conductance	6.7	57.8	25.2	32.0	29.0	57.4

Free energy differences were calculated from concentration gradients:[12]

$$\Delta G_{ion}(\mathbf{r}) = k_{\mathrm{B}}\, T\, N_{\mathrm{A}}\, \ln\left(\frac{\overline{\rho_{ion}}}{\rho_{ion}(\mathbf{r})}\right) \tag{1}$$

with the difference in free energy of the investigated cation $\Delta G_{ion}(\mathbf{r})$, the temperature of the simulation T, Boltzmann constant k_{B}, Avogadro number N_{A}, and the natural logarithm of the ratio between the average cation concentration in bulk water $\overline{\rho_{ion}}$ and the cation concentrations at the point of interest $\rho_{ion}(\mathbf{r})$. We assume that the channel pore is cylindrical symmetric, so $G_{ion}(\mathbf{r})$ becomes $G_{ion}(z, r)$ at pore axis z and radius r.

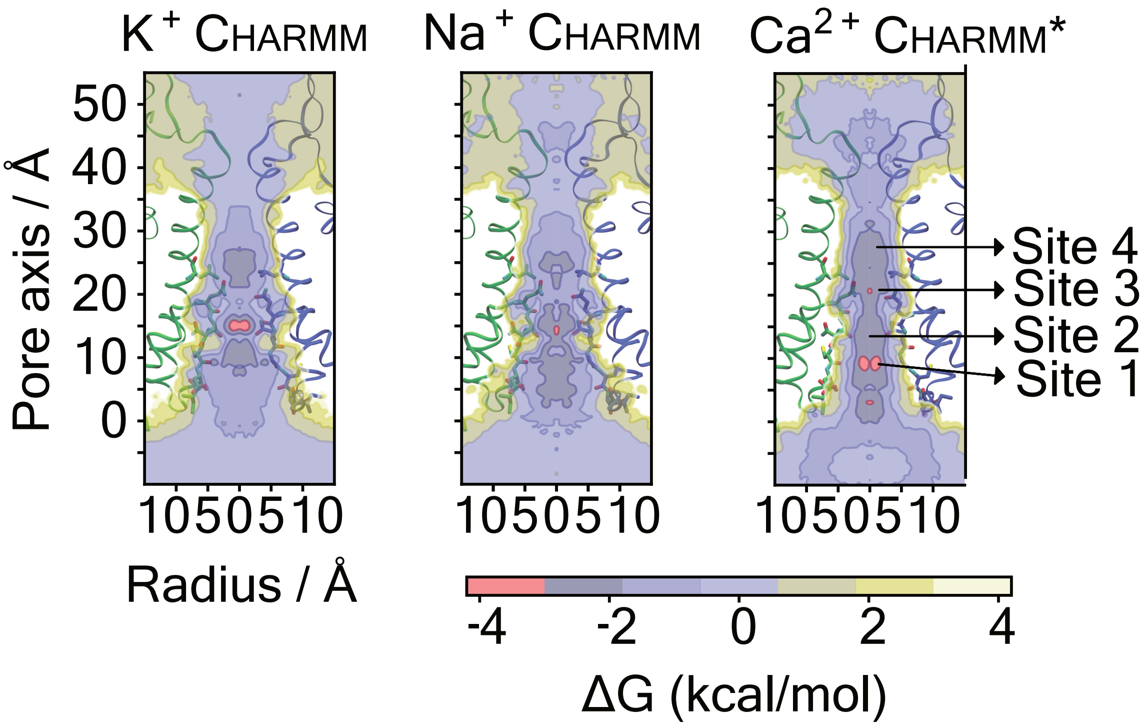

Figure S1: Two-dimensional ion occupancy within the selectivity filter resolved radially and along the pore axis (z-axis) as a contour plot calculated from MD simulations of GluA2 with different monovalent ions (K^+ and Na^+[13]) and divalent Ca^{2+}. The simulations were performed at 303 K using an imbalance of six cations between the two computational electrophysiology compartments. Ion concentration is plotted as free energy.

QM/MM simulations

The MiMiC interface[14–16] was used. It couples Gromacs[5] and Cpmd.[17] A representative snapshot of the low conductance mode observed during the force field based MD simulations served as starting structure. The QM part comprises a calcium ion located at 8 Å along the pore axis together with its first hydration shell and was treated with Density Functional Theory (DFT) at the BLYP[18,19] level in combination with empirical DFT-D2[20] van der Waals corrections. Core electrons were described by normconserving Troullier-Martins pseudopotentials,[21] while the valence electrons along with the 3*s* and the 3*p* states for the calcium ion were expanded in a set of plane waves up to a cutoff of 100 Ry. The cubic QM simulation box (edge length: 13.8 Å) was treated as an isolated system without periodic boundary conditions.[22] The MM part of the system was described by the same force field as in the classical MD simulations, *i.e.*, Charmm36m[6] and the Tip3p water model.[3]

The short range electrostatic interactions between the QM partition and any MM atom within 26.5 Å from the QM region were computed directly from the electronic density. The long range interactions for atoms more than 26.5 Å away from the QM region were calculated from a fifth-order multipole expansion of the QM electrostatic potential.[23] All van der Waals interactions were computed with Gromacs.[5]

Constant temperature simulations were carried out at 303 K using a Nosé-Hoover thermostat[24,25] with a chain length of four and a frequency of 4000 cm^{-1}. The Born-Oppenheimer Molecular Dynamics (BOMD) scheme was used with a timestep of 0.5 fs. The system underwent simulated annealing followed by a linear re-heating before the production phase (42 ps in total, see below for details).

QM/MM solvent exchange

The experimental water exchange time of $Ca^{2+}{}_{aq}$ is in the order of several nanoseconds.[26] *Ab initio* MD simulations report water exchange events in the range of a few dozen pico-

seconds.[27] In our QM/MM simulations of the complex in the channel, solvent exchange is faster by another order of magnitude, *i.e.*, occurs within a few picoseconds. The difference can be caused by (i) the fact that the complex is not in bulk water like in the previous calculations[27] or (ii) the QM description of ${Ca^{2+}}_{aq}$ on one side and the MM description of the surrounding water molecules on the other:[28] the Ca^{2+} binding distances for MM water molecules are shorter than the ones calculated at the DFT level (fig. S3). Here, we used our QM/MM approach to compare structural properties of the calcium-water complex with that of the force field in a time range for which there is no water exchange.

We propose a free energy profile with three distinct states (fig. S2). Different coordination environments characterize each state: (i) n QM (ii) n QM + 1 MM, and (iii) $(n + 1)$ QM water molecules ($n = 7$).

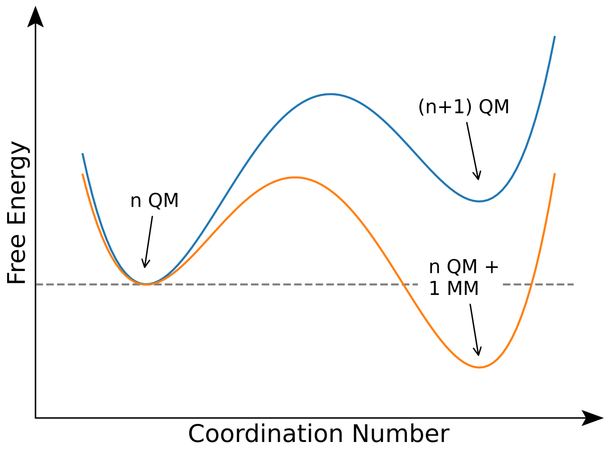

Figure S2: Proposed free energy profile sketch for QM/MM solvent exchange.

During the QM/MM production run dynamics, a MM water molecule enters the first hydration shell after a few picoseconds (fig. S3), going from state (i) to the energetically favorable state (ii). The level of description for an atom does not change automatically in the non-adaptive QM/MM scheme employed in this work.[14–16] Manually adjusting the level of description for the additional MM water molecule to QM corresponds to the transition from (ii) to (iii). Subsequently, one of the meanwhile eight QM water molecules disassociates from Ca^{2+} ((iii) $\rightarrow$ (i)), making room for the next MM water molecule to disturb the first

solvation sphere (fig. S3).

With an educated guess for the exchange rate constants ($k_{\mathrm{sim}} \approx 0.2\,\mathrm{ps}^{-1}$ for the QM/MM simulations and $k_{\mathrm{exp}} \approx 0.2\,\mathrm{ns}^{-1}$ as experimental value[26]), the difference in free energies of activation $\Delta\Delta G^{\#}$ between experiment ($\Delta G^{\#}_{\mathrm{exp}}$) and simulation ($\Delta G^{\#}_{\mathrm{sim}}$) can be estimated using the Eyring equation:[29]

$$
\begin{aligned}
(i) \quad & k_{\mathrm{sim}} = A \ \exp\left(-\frac{\Delta G^{\#}_{\mathrm{sim}}}{RT}\right) \\
(ii) \quad & k_{\mathrm{exp}} = \widetilde{A} \ \exp\left(-\frac{\Delta G^{\#}_{\mathrm{exp}}}{RT}\right) \\
(i/ii) \quad & \Delta\Delta G^{\#} = \Delta G^{\#}_{\mathrm{exp}} - \Delta G^{\#}_{\mathrm{sim}} = \log\left(\frac{k_{\mathrm{sim}}}{k_{\mathrm{exp}}}\right) RT = 4\,\mathrm{kcal/mol}
\end{aligned}
$$

with the universal gas constant R, temperature $T = 300\,\mathrm{K}$, assuming equal prefactors A and $\widetilde{A}$. The same reasoning with observations from full QM simulations ($k_{\mathrm{sim}} \approx 0.06\,\mathrm{ps}^{-1}$ [27]) yields $\Delta\Delta G^{\#} = 3\,\mathrm{kcal/mol}$. The difference in free energies of activation can therefore mostly be ascribed to limitations of DFT.

From the 10 QM/MM production run replicas that were carried out those parts that are suited for further analysis were identified from Figure S3. Skipping the first picosecond of each run for equilibration leaves the following time spans:

- Replica 1: ps 1 to 5 ; 11 to 14
- Replica 2: ps 1 to 4
- Replica 3: ps 1 to 4
- Replica 4: ps 1 to 7
- Replica 5: ps 1 to 3
- Replica 6: none
- Replica 7: none

- Replica 8: ps 1 to 7
- Replica 9: ps 1 to 9
- Replica 10: ps 1 to 8

This corresponds to a total of 42 ps QM/MM production phase.

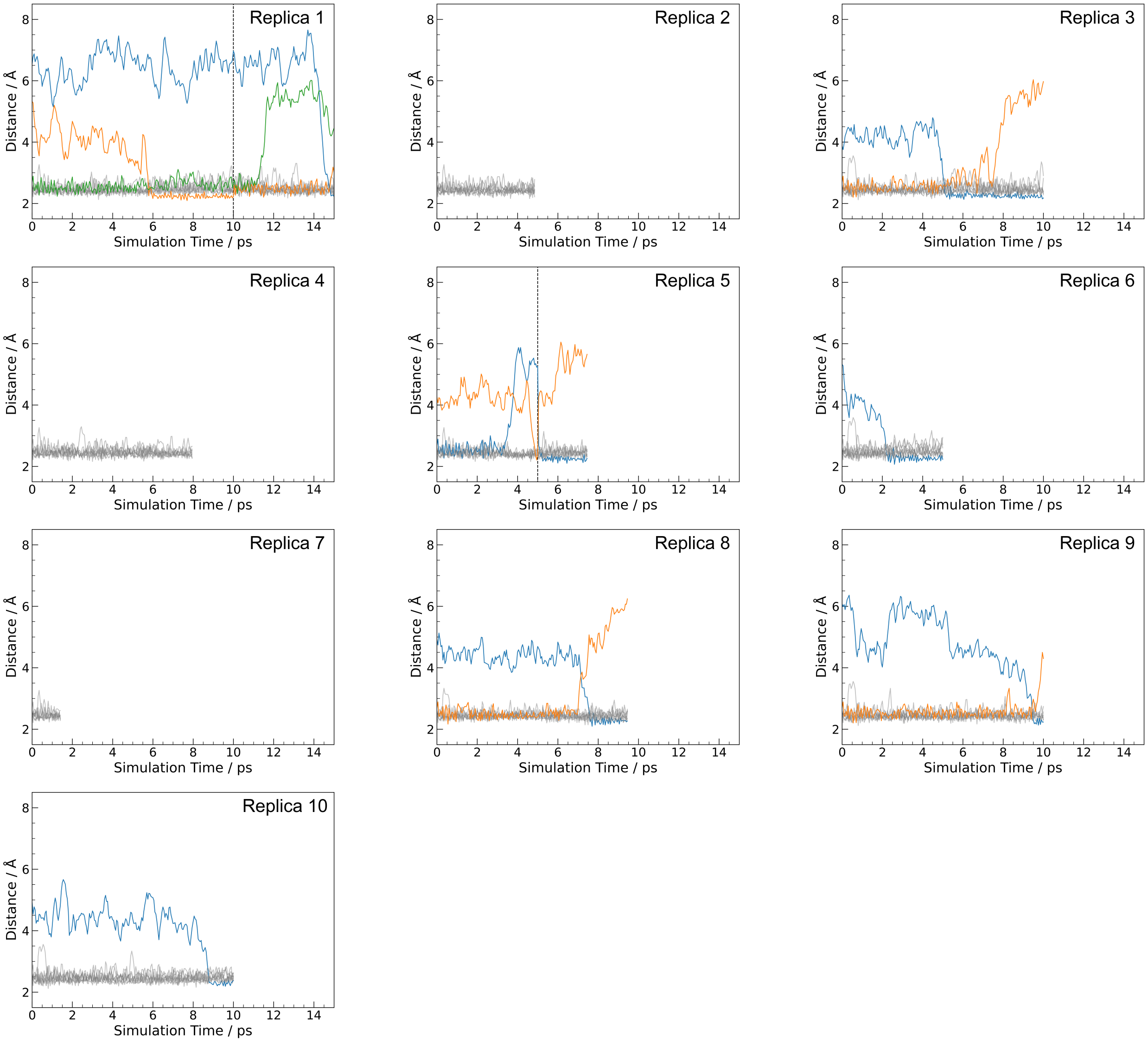

Figure S3: Distances between calcium(II) in the main binding site and oxygen atoms of coordinated water molecules during all ten production run replicas. At the beginning of each run, the seven water molecules that form the first hydration shell are included in the QM partition. Water molecules that change between first and second hydration shell are shown in colored lines, all remaining in grey. Vertical black dashed lines indicate an adjustment of the QM/MM partitioning.

Calcium binding profile in an AMPAR pore mimic

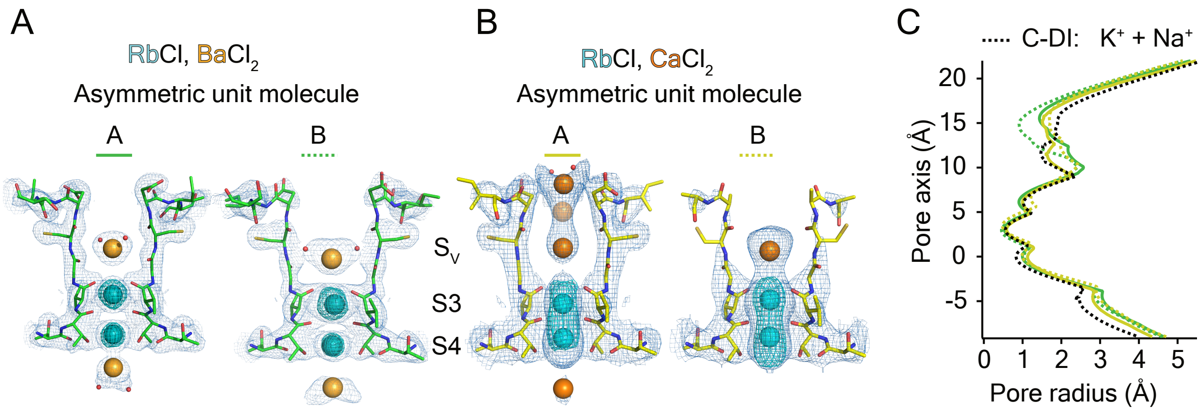

Figure S4: The 2F$_o$-F$_c$ electron density maps (blue mesh) of NaK C-DI, contoured at 1σ, show (A) Ba^{2+} (pale orange) and Rb$^+$ (cyan) (PDB ID: 8AYP) as well as (B) Ca^{2+} (orange) and Rb$^+$ (PDB ID: 8AYQ) binding profiles in the selectivity filter. Water molecules are shown in red. The lower occupancy alternate conformation of the uppermost Ca^{2+} ion (molecule A) is highlighted by decreased transparency. Anomalous difference density for Rb$^+$ is shown in cyan, contoured at 3σ. (C) Pore profiles of the different ion complexes and asymmetric units of NaK C-DI, generated with Hole.[30] The origin is set to the plane of hydroxyl oxygen of T63.

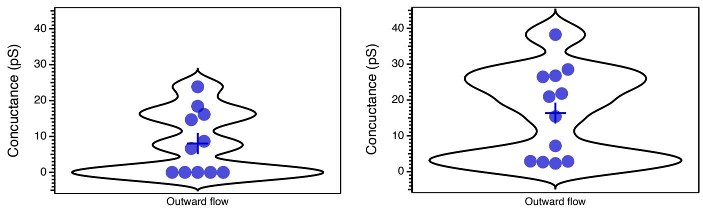

Figure S5: Simulated outward Ca^{2+} conductance of NaK C-DI for each 500 ns simulation run at a transmembrane voltage of 200 mV (left) and 400 mV (right), respectively. Mean of the conductance is shown as cross. The simulations were performed using computational electrophysiology setup with the Charmm36m force field[6] and the multi-site Ca^{2+} model.[10]

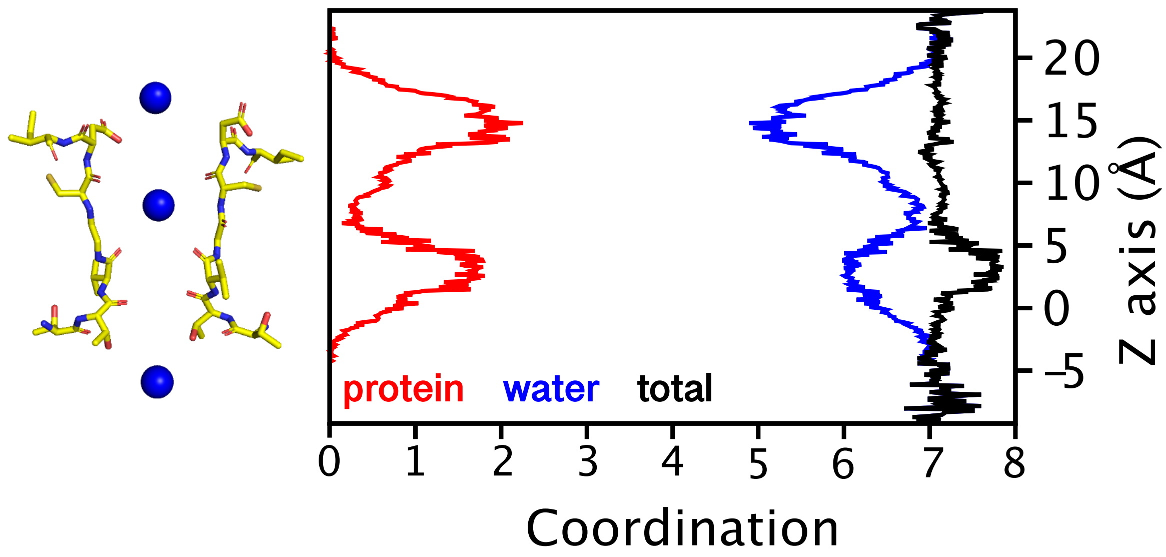

Figure S6: The number of ion-interacting oxygens within the first hydration shell in the selectivity filter region along the pore axis derived from Ca^{2+} permeation simulations. The simulations were performed using the computational electrophysiology setup with the CHARMM36M force field[6] and the multi-site Ca^{2+} model.[10] The number of coordinating water oxygens is shown in blue, the number of coordinating protein oxygens is in red, and their sum in black.

Table S3: **Data collection and refinement statistics.** Data for the highest resolution shell are given in parentheses.

**Data collection**	**Rb^+/ Ca^{2+} (8AYQ)**	**Rb^+/ Ba^{2+} (8AYP)**
Space group	$C222_1$	$C222_1$
Cell dimensions		
a, b, c (Å)	67.6 175.5 67.3	68.2 177.5 68.0
α, β, γ (°)	90.0 90.0 90.0	90.0 90.0 90.0
Wavelength (Å)	0.815	0.918
Resolution range (Å)	33.78 - 2.75 (2.85 - 2.75)	44.68 - 2.10 (2.17 - 2.10)
Redundancy	2.1 (2.0)	4.4 (4.5)
Completeness (%)	96.0 (92.3)	99.4 (98.4)
R_{pim}	0.13 (0.87)	0.06 (0.77)
$I/\sigma I$	4.1 (0.6)	9.0 (0.9)
$CC_{1/2}$	0.99 (0.40)	0.99 (0.39)
**Refinement**		
Resolution range (Å)	33.78 - 2.75 (2.85 - 2.75)	44.68 - 2.10 (2.17 - 2.10)
No. of reflections	10354 (980)	24482 (2383)
R_{work}/ R_{free} (%)	25.3 / 29.5	22.6 / 26.7
No of atoms	3084	3158
Protein	2892	2845
Ligands	407	240
Water	37	73
Average *B* factors ($Å^2$)	89.04	60.20
Protein	88.65	59.26
Ligands	101.01	69.27
Water	69.22	66.93
RMSD		
Bond lengths (Å)	0.007	0.004
Bond angles (°)	1.16	0.66
Ramachandran plot (%)		
most favored	98.08	97.74
allowed	1.92	2.26
outliers	0.00	0.00
Rotamer outliers (%)	0.00	0.00
Clashscore	10.16	6.19

References

(1) Twomey, E. C.; Yelshanskaya, M. V.; Grassucci, R. A.; Frank, J.; Sobolevsky, A. I. Channel opening and gating mechanism in AMPA-subtype glutamate receptors. *Nature* **2017**, *549*, 60–65.

(2) Wu, E. L.; Cheng, X.; Jo, S.; Rui, H.; Song, K. C.; Dávila-Contreras, E. M.; Qi, Y.; Lee, J.; Monje-Galvan, V.; Venable, R. M.; Klauda, J. B.; Im, W. CHARMM-GUI membrane builder toward realistic biological membrane simulations. *J. Comput. Chem.* **2014**, *35*, 1997–2004.

(3) Jorgensen, W. L.; Chandrasekhar, J.; Madura, J. D.; Impey, R. W.; Klein, M. L. Comparison of simple potential functions for simulating liquid water. *J. Chem. Phys.* **1983**, *79*, 926–935.

(4) Kutzner, C.; Grubmüller, H.; De Groot, B. L.; Zachariae, U. Computational electrophysiology: The molecular dynamics of ion channel permeation and selectivity in atomistic detail. *Biophys. J.* **2011**, *101*, 809–817.

(5) Abraham, M. J.; Murtola, T.; Schulz, R.; Páll, S.; Smith, J. C.; Hess, B.; Lindah, E. Gromacs: High performance molecular simulations through multi-level parallelism from laptops to supercomputers. *SoftwareX* **2015**, *1-2*, 19–25.

(6) Huang, J.; MacKerell, A. D. J. CHARMM36 all-atom additive protein force field: validation based on comparison to NMR data. *J Comput Chem* **2013**, *34*, 2135–2145.

(7) Berendsen, H. J. C.; Postma, J. P. M.; van Gunsteren, W. F.; DiNola, A.; Haak, J. R. Molecular dynamics with coupling to an external bath. *J. Chem. Phys.* **1984**, *81*, 3684–3690.

(8) Hess, B.; Bekker, H.; Berendsen, H. J. C.; Fraaije, J. G. E. M. LINCS: A linear constraint solver for molecular simulations. *J. Comput. Chem.* **1997**, *18*, 1463–1472.

(9) Darden, T.; York, D.; Pedersen, L. Particle mesh Ewald: An N˙log(N) method for Ewald sums in large systems. *J. Chem. Phys.* **1993**, *98*, 10089–10092.

(10) Zhang, A.; Yu, H.; Liu, C.; Song, C. The Ca^{2+} permeation mechanism of the ryanodine receptor revealed by a multi-site ion model. *Nat. Commun.* **2020**, *11*, 1–10.

(11) Bussi, G.; Donadio, D.; Parrinello, M. Canonical sampling through velocity rescaling. *J. Chem. Phys.* **2007**, *126*.

(12) Boltzmann, L. In *Wissenschaftliche Abhandlungen*; Hasenöhrl, F., Ed.; Cambridge Library Collection - Physical Sciences; Cambridge University Press, 2012; Vol. 1; pp 49–96.

(13) Biedermann, J.; Braunbeck, S.; Plested, A. J.; Sun, H. Nonselective cation permeation in an AMPA-type glutamate receptor. *Proc. Natl. Acad. Sci. U.S.A.* **2021**, *118*, 1–11.

(14) Bolnykh, V.; Olsen, J. M. H.; Meloni, S.; Bircher, M. P.; Ippoliti, E.; Carloni, P.; Rothlisberger, U. Extreme Scalability of DFT-Based QM/MM MD Simulations Using MiMiC. *J. Chem. Theory Comput.* **2019**, *15*, 5601–5613.

(15) Olsen, J. M. H.; Bolnykh, V.; Meloni, S.; Ippoliti, E.; Bircher, M. P.; Carloni, P.; Rothlisberger, U. MiMiC: A Novel Framework for Multiscale Modeling in Computational Chemistry. *J. Chem. Theory Comput.* **2019**, *15*, 3810–3823.

(16) Bolnykh, V.; Olsen, J. M. H.; Meloni, S.; Bircher, M. P.; Ippoliti, E.; Carloni, P.; Rothlisberger, U. MiMiC: Multiscale Modeling in Computational Chemistry. *Front. Mol. Biosci.* **2020**, *7*, 45.

(17) Hutter, J.; Alavi, A.; Deutsch, T.; Bernasconi, M.; Goedecker, S.; Marx, D.; Tuckerman, M.; Parrinello, M. CPMD, Copyright IBM Corp 1990-2022, Copyright MPI für Festkörperforschung Stuttgart 1997-2001.

(18) Becke, A. D. Density-functional exchange-energy approximation with correct asymptotic behavior. *Phys. Rev. A* **1988**, *38*, 3098–3100.

(19) Lee, C.; Yang, W.; Parr, R. G. Development of the Colle-Salvetti correlation-energy formula into a functional of the electron density. *Phys. Rev. B* **1988**, *37*, 785–789.

(20) Grimme, S. Accurate description of van der Waals complexes by density functional theory including empirical corrections. *J. Comput. Chem.* **2004**, *25*, 1463–1473.

(21) Troullier, N.; Martins, J. L. Efficient pseudopotentials for plane-wave calculations. *Phys. Rev. B* **1991**, *43*, 1993–2006.

(22) Martyna, G. J.; Tuckerman, M. E. A reciprocal space based method for treating long range interactions in ab initio and force-field-based calculations in clusters. *J. Chem. Phys.* **1999**, *110*, 2810–2821.

(23) Laio, A.; VandeVondele, J.; Rothlisberger, U. A Hamiltonian electrostatic coupling scheme for hybrid Car-Parrinello molecular dynamics simulations. *J. Chem. Phys.* **2002**, *116*, 6941–6947.

(24) Nosé, S. A unified formulation of the constant temperature molecular dynamics methods. *J. Chem. Phys.* **1984**, *81*, 511–519.

(25) Hoover, W. G. Canonical dynamics: Equilibrium phase-space distributions. *Phys. Rev. A* **1985**, *31*, 1695–1697.

(26) Helm, L.; Merbach, A. E. Water exchange on metal ions: Experiments and simulations. *Coordin. Chem. Rev.* **1999**, *187*, 151–181.

(27) Bogatko, S.; Cauët, E.; Bylaska, E.; Schenter, G.; Fulton, J.; Weare, J. The aqueous Ca^{2+} system, in comparison with Zn2+, Fe3+, and Al3+: An ab initio molecular dynamics study. *Chem. - Eur. J.* **2013**, *19*, 3047–3060.

(28) Watanabe, H. C.; Cui, Q. Quantitative Analysis of QM/MM Boundary Artifacts and Correction in Adaptive QM/MM Simulations. *J. Chem. Theory Comput.* **2019**, *15*, 3917–3928.

(29) Eyring, H. The activated complex in chemical reactions. *J. Chem. Phys.* **1935**, *3*, 63–71.

(30) Smart, O. S.; Neduvelil, J. G.; Wang, X.; Wallace, B.; Sansom, M. S. HOLE: A program for the analysis of the pore dimensions of ion channel structural models. *J. Mol. Graphics* **1996**, *14*, 354–360.
